# RETRACTED: Schiavello et al. Identification of Redox-Sensitive Transcription Factors as Markers of Malignant Pleural Mesothelioma. *Cancers* 2021, *13*, 1138

**DOI:** 10.3390/cancers18162564

**Published:** 2026-08-10

**Authors:** Martina Schiavello, Elena Gazzano, Loredana Bergandi, Francesca Silvagno, Roberta Libener, Chiara Riganti, Elisabetta Aldieri

**Affiliations:** 1 Department of Medical Sciences, University of Torino, 10126 Turin, Italy; martina.schiavello@unito.it; 2 Department of Life Sciences and Systems Biology, University of Torino, 10135 Turin, Italy; elena.gazzano@unito.it; 3 Interdepartmental Center for Studies on Asbestos and Other Toxic Particulates “G. Scansetti”, University of Torino, 10126 Turin, Italy; chiara.riganti@unito.it; 4 Department of Oncology, University of Torino, 10126 Turin, Italy; loredana.bergandi@unito.it (L.B.); francesca.silvagno@unito.it (F.S.); 5 Department of Integrated Activities Research and Innovation, Azienda Ospedaliera SS. Antonio e Biagio e Cesare Arrigo, 15121 Alessandria, Italy; rlibener@ospedale.al.it

The journal retracts the article titled “Identification of Redox-Sensitive Transcription Factors as Markers of Malignant Pleural Mesothelioma” [1], cited above.

Following publication, concerns were brought to the attention of the Editorial Office regarding the integrity of a number of figures presented in this article [1].

In accordance with the journal’s standard procedures, an investigation was conducted by the Editorial Office and Editorial Board which identified indications of inappropriate image editing and duplication in Figures 1A, 3A and 4C. Although the authors cooperated with the investigation, the explanations and supporting materials provided were not deemed sufficient by the Editorial Board to resolve the identified concerns. As a result, the Editorial Board has lost confidence in the reliability of the findings and has decided to retract this publication [1], as per MDPI’s retraction policy.

This retraction was approved by the Editor-in-Chief of the journal *Cancers*.

The authors have been informed of this retraction and have indicated their disagreement with this decision.
